# Metabolic imbalance driving immune cell phenotype switching in autoimmune disorders: Tipping the balance of T‐ and B‐cell interactions

**DOI:** 10.1002/ctm2.1626

**Published:** 2024-03-18

**Authors:** Matteo Barberis, Alejandra Rojas López

**Affiliations:** ^1^ Molecular Systems Biology School of Biosciences Faculty of Health and Medical Sciences University of Surrey Guildford Surrey UK; ^2^ Centre for Mathematical and Computational Biology, CMCB University of Surrey Guildford Surrey UK; ^3^ Synthetic Systems Biology and Nuclear Organization Swammerdam Institute for Life Sciences University of Amsterdam Amsterdam The Netherlands

**Keywords:** ageing, autoimmune disorders, B‐cell phenotypes, metabolic disorders, metabolic switches, systems immunology, T‐cell phenotypes

## Abstract

The interplay between the immune system and the metabolic state of a cell is intricate. In all phases of an immune response, the corresponding metabolic changes shall occur to support its modulation, in addition to the signalling through the cytokine environment and immune receptor stimulation. While autoimmune disorders may develop because of a metabolic imbalance that modulates switching between T‐cell phenotypes, the effects that the interaction between T and B cells have on one another's cellular metabolism are yet to be understood in disease context. Here, we propose a perspective which highlights the potential of targeting metabolism to modulate T‐ and B‐cell subtypes populations as well as T–B and B–T cell interactions to successfully treat autoimmune disorders. Specifically, we envision how metabolic changes can tip the balance of immune cells interactions, through definite mechanisms in both health and disease, to explain phenotype switches of B and T cells. Within this scenario, we highlight targeting metabolism that link inflammation, immunometabolism, epigenetics and ageing, is critical to understand inflammatory disorders. The combination of treatments targeting immune cells that cause (T/B) cell phenotype imbalances, and the metabolic pathways involved, may increase the effectiveness of treatment of autoimmune disorders, and/or ameliorate their symptoms to improve patients’ quality of life.

## Graphical abstract

1. Metabolic imbalance underlies development of autoimmune disorders.
2. Metabolic changes modulate switching between immune cell phenotypes.
3. Targeting metabolism regulates T–B and B–T cell interactions.
4. Ageing at the interface between autoimmune disorders and metabolic syndromes.

Conceptual scheme of T–B and B–T cell interactions at the metabolic level. The mutual modulation of metabolic pathways between T and B cells favours an increased metabolic activity, generating feedback of activation. T cells can affect B‐cell metabolism and B cells can affect T‐cell metabolism, through the signalling pathways triggered both by receptor stimulation and cytokine release. Through mutual regulation, T and B cells can meet the metabolic demand required in each stage of their development and differentiate to a specific phenotype.

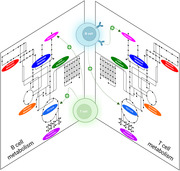

## 1 INTRODUCTION

The immune system is a complex, non‐linear network of molecules that work together to protect an organism against harmful (extracellular and intracellular) cues. The cooperation and interaction between the lymphocytes T and B (referred to as T and B cells) through multiple mechanisms is recognised as a key element of adaptive immunity at different levels. Specifically, this interaction is required to select immune cells in response to pathogens or dangerous self‐cells, and to promote differentiation of immune cells into the appropriate phenotype considering the required organismal response.^1^ The established literature shows the interaction between T and B cells (referred here to as T–B or B–T cell interactions) to be mediated by receptor interactions and cytokines, the signalling pathways they trigger and, ultimately, by the response activated at the gene expression level. Another relevant, yet not fully understood, mediators of lymphocyte interactions are the responses elicited at the metabolic level, which affects the immune cell state. Indeed, to execute any immune program and develop a certain cell phenotype, an interplay is required between appropriate signalling and transcription responses and the cell's metabolic state to guarantee the required availability of building molecules and energy levels for cellular homeostasis.^2^, ^3^

Within this scenario, it is critical to address the role of metabolic changes mediated by T cells on B‐cell metabolism and, vice versa, by B cells on T‐cell metabolism in the immune response. An example of the former is the interaction of a T cell with a naïve B cell through the major histocompatibility complex (MHC) class II molecules; it promotes activation of the protein kinase B/mammalian target of rapamycin (Akt/mTOR) pathway, which in turn modulates the B‐cell metabolism by switching the metabolic flux from fatty acid oxidation and oxidative phosphorylation (OXPHOS) to glycolysis and tricarboxylic acid (TCA) cycle.^4^ This change allows for faster energy production and activation of anaplerotic reactions to produce the metabolites required in biosynthetic pathways. The mTOR pathway can be activated by various signals, including T‐cell receptor (TCR) and Toll‐like receptor (TLR) signals, insulin and Notch 1 stimulation. The net effect of mTOR signalling depends on the cellular context; however, a tight pathway regulation is required for naïve cells to maintain a quiescent state, while its excessive inhibition can lead to anergy.^5^

Conversely, an example of the modulation of T‐cell metabolism by B cells is the B‐cell‐mediated T‐cell activation during insulin resistance. In visceral adipose tissue (VAT) of mice, immune cell infiltration of macrophages and T cells are involved in chronic inflammation. The abundance of fatty acids promotes B cells’ class switching to immunoglobulin (IgG), antibody production and interferon‐γ (IFN‐γ) production. These B cells exhibit an increased glucose metabolism and can modulate Th1/Th2/Treg ratios, favouring pro‐inflammatory phenotypes.^6^ B cells also contribute to the activation of CD8+ cytotoxic cells, which along with antibody production can affect pancreas cells and cause insulin resistance. In addition, Th1 cells can also aid class switching in B cells, resulting in a feedback loop in which B and T cells activate one another at the metabolic level, increasing their glycolytic flux and protein turnover, ultimately causing chronic inflammation.^6^

In the disease context, there is evidence that a prolonged exposure to certain cytokines contributes to the metabolic rewiring of lymphocytes. For example, it has been observed that response to TCR stimulation and IFN‐α signals affects the metabolic capacity of CD8+ T cells from Systemic Lupus Erythematosus (SLE) patients. These cells exhibit enlarged mitochondria, downregulation of mitochondria‐derived genes and a lower respiratory capacity.^7^ Also, T cells from healthy donors can develop this abnormal phenotype seen in SLE upon TCR stimulation and IFN‐α exposure.^7^ Considering that a higher IFN‐α expression correlates with disease severity, the proportion of T‐cell subtypes may affect disease progression and inform on the severity of the disease course.

Overall, distinct metabolic processes control the function of T‐cell phenotypes in autoimmune disorders, with an imbalance towards Th1/Th17 phenotypes to the detriment of the regulatory T‐cell (Treg) phenotype, resulting in more inflammatory responses.^1^, ^8^ In healthy individuals, these are necessary to clear infections; however, when in excess or directed towards non‐pathogenic antigens, they may lead to autoinflammatory or autoimmune disorders. Here, we critically discuss the metabolic requirements for T and B cells in health versus disease states and propose how the interaction between these immune cells governs the metabolic switches driving T/B‐cell phenotype imbalance in autoimmune disorders.

## 2 T‐CELL METABOLISM IN HEALTH AND DISEASE

**FIGURE 1:**
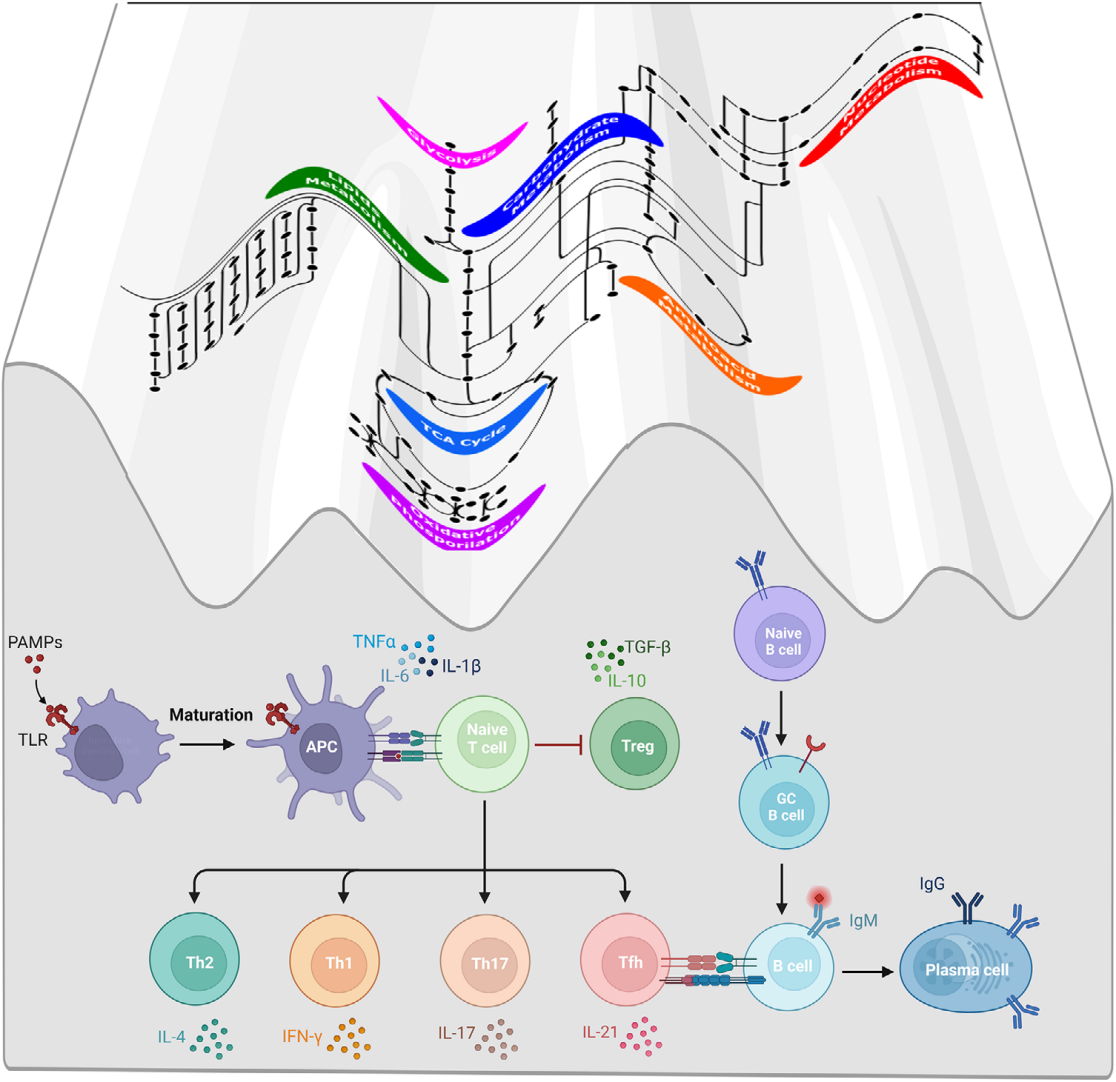
The metabolic state shapes the landscape of T‐ (and B‐) cell phenotypes. Metabolic changes, along with the cytokine environment, drive T (and B) cell differentiation into various phenotypes depending on the availability of energy. For instance, T cells require an increased glycolytic flux to move through the phenotype landscape towards the T helper (Th) subtypes Th1, Th2, Th17, regulatory T‐cell (Treg) and T follicular helper (Tfh). In addition, the metabolic activation of T cells disfavours Treg phenotype while inducing inflammatory subtypes such as Th1 and Th17. B cells also need energy for their activation, as well as for the building blocks used for antibody production, resulting in the metabolic state affecting the interactions between B and T cells (in particular with Tfh cells). Created with and adapted from BioRender.com.

The importance of CD4^+^ T‐cell phenotypes, particularly the T follicular helper (Tfh) phenotype, to drive the immune response in health versus disease is well‐established.^1^ Homeostasis between pro‐ and anti‐inflammatory phenotypes is key to responding timely against immune challenges and avoiding exacerbated or abnormal inflammation. To accomplish this delicate balance, T cells must (1) sense environmental cues, such as nutrient availability, chemokines, growth factors and other signalling molecules, and (2) switch among different metabolic states. This concept is illustrated in Figure 1. The main connections between signalling and metabolic pathways of T cells in health and disease are indicated in Table 1. For example, the switch from OXPHOS to glycolysis represents the main energy‐producing route, to meet the metabolic demands necessary to switch from a cell's quiescent state to a proliferative state. Zooming in, TCR stimulation and interleukin‐7 (IL‐7) modulate glucose metabolism.^9^ On the one hand, TCR activation triggers the mitogen‐activated protein kinase/phosphatidylinositol 3‐kinase/protein kinase B (MAPK/PI3K/Akt) signalling pathway, which in turn promotes the expression of the glucose transporter (GLUT) protein Glut1. On the other hand, IL‐7, which is produced by cells in the T‐cell zone of lymphoid organs, among others, promotes metabolic flux through glycolysis. By doing so, TCR and IL‐7 are involved in the development and survival of T cells and modulate T‐cell homeostasis. However, the proliferative response must be controlled, to avoid the (unwanted) development of autoimmune responses. To realise this, molecules such as the B‐cell lymphoma 2 (Bcl‐2) interacting mediator of cell death (Bim) that are related to mitochondrial apoptosis can modulate the metabolic and signalling environment by inducing apoptosis in lymphocytes. This way, the proliferation response would cease, and any potential inflammatory signals would disappear. Furthermore, another cue for proliferation termination is the disappearance of the inflammatory cytokine IL‐2. In the absence of enough TCR co‐stimulation, the PI3K/Akt pathway becomes inactivated, leading to Glut1 degradation via the proteasome. Thus, a T cell shall switch the metabolic program to guarantee cellular survival as well as shall restrict its metabolic activity when required. In fact, abnormal metabolic activity can trigger the development of autoimmune disorders, as T cells may become overactivated.^9^

**TABLE 1:** Connections between signalling and metabolic pathways of immune cells, with examples of how these participate in processes related to health and disease.

Cells	Signals	Signalling pathways	Metabolic pathways	Health conditions	Disease imbalance
**T cell**					
Th cells	TCR activation IL‐7	MAPK/PI3K/Akt	OXPHOS Glycolysis	Switch from OXPHOS to glycolysis and increased glucose transport via GLUT1^9^	
Th cells	Bim IL‐2 absence	MAPK/PI3K/Akt inactivation		Mitochondrial apoptosis to control T‐cell population^9^	
Th cells	HIF‐1α Myc	mTOR	Glycolysis	Proteasome inhibition, T‐cell activation, proliferation and differentiation^10^	Treg/Th1/Th17 imbalance in HT^18^
Th cells	O_2_^–^, H_2_O_2_, HClO		ROS metabolism Glycolysis	Limit inflammatory response by inhibiting T‐cell clonal expansion^11^	
Th1 and CD8+ cytotoxic T cells					Infiltration of immune cells in exocrine glands in SS^15^
Th17Treg	STAT3 HIF‐1α	PI3K/Akt mTOR	ROS metabolism Glycolysis OXPHOS	Promotes B‐cell expansion favouring the Th17 phenotype over the Treg phenotype. Treg/Th1 balance^16^	In SLE, overactivation of mTOR and ROS exacerbates the mechanism^14^
Treg	FOXP3 IL‐10			Favours Treg phenotype over pro‐inflammatory phenotypes^17^	
**B cell**					
B cells	HIF‐1α IL‐4	mTOR		Dynamic oxygen level allows for B‐cell development, activation and differentiation^22^, ^23^	
Pre‐B cells	IL‐7		Glycolysis Fatty acids, amino acids and protein synthesis		
GC B cells	TNF‐α IFN‐γ	cGAS‐STING	Glycolysis ROS metabolism	Metabolic switch needed for B‐cell development and activation^21^	SLE, RA, MS, overreactive B cells showing mitochondrial dysfunction, increased death‐inducing signals^28^, ^29^, ^30^
Naïve and memory B cells	BAFF TNF‐α	AMPK mTOR	Fatty acid oxidation OXPHOS	Autophagy allows cells to meet minimal metabolic demands and survive with limited growth signals^22^, ^31^	Exacerbated mTORC1 activation, observed in SLE and RA^22^, ^31^
Breg	IL‐10 IL‐35			Suppress differentiation of inflammatory Th phenotypes^26^	
**B–T and T–B cell interactions**					
B–T cells	Myc Bcl‐2	mTOR/Akt	Biosynthetic pathways Glycolysis OXPHOS	Appropriate Myc signalling balances the activation or apoptotic death of T cells^34^, ^35^	Hyperactivation of T cells by B cells leads to T cells exhaustion and chronic inflammation^6^, ^42^
Tfh–B cell	Bcl‐6 CXC‐R5 IL‐21	PI3K/Akt mTOR	Glycolysis Fatty acids, amino acids and protein synthesis ROS metabolism	Tfh cells induce metabolic switch on B cell from GC, enhancing metabolic activity^12^	Dysregulation of GC formation, increased B‐cell activation, antibody production, autoimmunity MS, SLE, RA, etc.^14^, ^37^
T–B cells	CD40L ICOS			Delivery of Th help and contribution to antibody production by B cells^39^	

*Note*: Cell types involved, signals (either soluble or receptor transduced), signalling and metabolic pathways, as well as examples of health and disease mechanisms (when known) are indicated.

Abbreviations: BAFF, B‐cell activating factor; Breg, regulatory B cell; CXC‐R5, C‐X‐C chemokine receptor type 5; GC, germinal center; HIF‐1α, hypoxia inducible factor 1α; HT, Hashimoto's thyroiditis; IFN‐γ, interferon‐γ; IL, interleukin; MS, Multiple Sclerosis; OXPHOS, oxidative phosphorylation; RA, Rheumatoid Arthritis; ROS, reactive oxygen species; SLE, Systemic Lupus Erythematosus; SS, Sjögren's syndrome; TCR, T‐cell receptor; Tfh, T follicular helper; Th, T helper; TNF‐α, tumour necrosis factor‐α; Treg, regulatory T cell.

An important aspect of T‐cell metabolism is the dynamic of protein synthesis and degradation, which contribute to the demand for energy and amino acids during T‐cell activation, proliferation and differentiation.^10^ Indeed, this process requires de novo mRNA synthesis and prioritisation of the proteins required at each stage. The balance between synthesis and degradation relies on proteasome inhibition via mTORC1 activation, which promotes hypoxia inducible factor 1α (HIF‐1α) expression that in turn results in Myc expression and increased glycolysis flux. In addition, regulation of protein synthesis has a direct crosstalk with amino acid metabolism, as mRNA translation is tightly regulated by the availability of amino acid charged tRNAs and the regulation of amino acid transporter expression.^10^

Another relevant aspect of the cell's metabolic state is the effect of the metabolic environment on lymphocyte differentiation. For example, an oxidative environment generated via the production of O_2_^–^, H_2_O_2_ and HClO by neutrophils inhibits the metabolic switch from mitochondrial respiration to aerobic glycolysis, which is required for T‐cell clonal expansion.^11^ This effect appears to be mediated by H_2_O_2_, which suppresses IFN‐γ production and lymphocyte proliferation. Although the underlying mechanism is not completely characterised yet, their role seems to be related to limiting the inflammatory response and therefore preventing autoimmunity.

Different CD4^+^ T‐cell phenotypes express different membrane markers, produce a characteristic cytokine profile, carry specific metabolic signatures, and have different functions. For example, Tfh cells promote the differentiation and function of B cells in the germinal center (GC), a specialised structure that emerges in follicles of secondary lymphoid organs after encountering a T‐cell‐dependent antigen. Tfh cells are characterised by the expression of the transcription factor B‐cell lymphoma 6 (Bcl‐6) and of the C‐X‐C chemokine receptor type 5 (CXC‐R5) and are key to developing B‐cell‐mediated humoral immunity; they are involved in B‐cell metabolic switching, promoting mTOR activation and, therefore, promoting energy production.^12^ Tfh cells expressing Bcl‐6 appear to have a crucial role in tissue homeostasis along with IL‐21, which is involved in the regulation of GC formation and is an important mediator of apoptosis. Indeed, experiments conducted in mice suggest that Bcl‐6 deletion results in a reduction in the number of GCs and of B‐cell activation.^13^

In the context of disease, dysregulation of Tfh cells or their imbalance compared to other T‐cell phenotypes may cause an abnormal B‐cell differentiation, resulting in the development of well‐known autoimmune disorders such as SLE, Rheumatoid Arthritis (RA) and Multiple Sclerosis (MS).^14^ T‐cell dysregulation occurs also in less‐studied immune disorder such as the Sjögren's syndrome (SS), a chronic autoimmune disorder characterised by xerostomia (dry mouth) and keratoconjunctivitis that is caused by lymphatic infiltration of exocrine glands. The infiltration is mediated by T helper cells with a Tfh‐like phenotype observed in both blood and salivary glands. Here, the epithelial cells of the salivary glands act as antigen‐presenting cells (APCs), recruiting IL‐1β‐producing macrophages, which in turn promote the activation of Th1 and CD8+ cytotoxic T cells. Despite the progression of SS is not as severe as other autoimmune disorders, it is associated with an increased prevalence of metabolic disorders; specifically, it is correlated with higher levels of inflammatory proteins that contribute to the development of insulin resistance.^15^ In addition, the increased levels of serum lipids appear to contribute to the development of subclinical cardiovascular diseases. Dyslipidaemia (lipids imbalance) and insulin resistance may in turn impact on the function of memory T cells, reducing the anergic state.^15^

**FIGURE 2:**
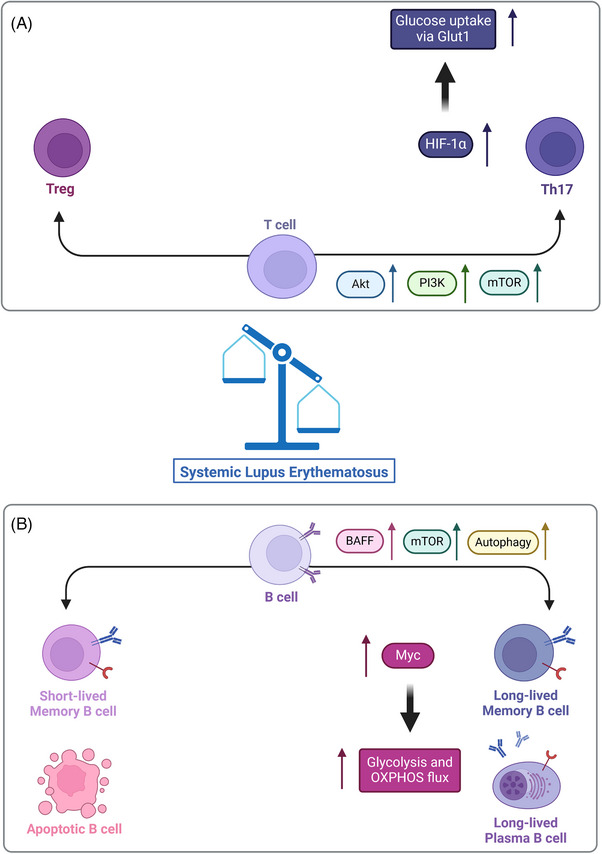
T‐ and B‐cell metabolic changes in the progression of autoimmune diseases, taking Systemic Lupus Erythematosus (SLE) as an example. In healthy conditions, a balance occurs between pro‐ and anti‐inflammatory B‐ and T‐cell phenotypes. These immune phenotypes have certain metabolic demands, therefore activation of signalling pathways during the immune response triggers specific metabolic changes. However, in autoimmune disorders, an imbalance in the immune cell subtypes is observed. Specifically, for SLE, increased signalling through the Akt/PI3K/mTOR axis promotes hypoxia inducible factor 1α (HIF‐1α) overexpression, resulting in an enhanced metabolic activity on T cells that favours the Th17 phenotype, with a high metabolic flux through glycolysis and TCA cycle, ultimately leading to an imbalance in the Th17/Treg ratio (A). On the other hand, increased metabolic activity in B cells, with high B‐cell activating factor (BAFF) levels, and enhanced autophagy, results in Myc overexpression with high glycolysis and oxidative phosphorylation (OXPHOS), thus promoting B‐cell survival and proliferation. Therefore, short‐lived cells and cells that should undergo apoptosis differentiate into long‐lived memory cells, as well as antibody‐producing cells or plasma cells, that are deeply involved in SLE pathogenesis and symptomatology (B). Created with and adapted from BioRender.com.

As mentioned in the examples above, oxidative stress and metabolic imbalance affect T‐cell phenotypes, potentially causing or contributing to autoimmune disorders. For example, excessive oxidative stress correlates with SLE development. Briefly, the excess of reactive oxygen species (ROS) triggers the PI3K/Akt pathway and mTORC1 activation, due to the hyperpolarisation of the mitochondrial membrane, which contributes to T‐cell expansion and promotes differentiation of T cells to the Th17 phenotype, to the detriment of the Treg phenotype.^16^ mTORC1 activation results in the phosphorylation of STAT3 and in an upregulation of the HIF‐1, which increases expression of the glucose transporter Glut1, thus glucose uptake, and the glycolytic flux (Figure 2A). Therefore, it appears that mitochondrial dysfunction may contribute to SLE development, resulting in a metabolic imbalance in tissues.^14^ In this context, the Treg phenotype is required to prevent undesired inflammation in tissues and assure the clearance of inflammation after resolving an infection. This anti‐inflammatory role becomes clearer in mucosal areas, as well as skin and other tissues where the organism is constantly in contact with innocuous non‐self‐antigens. For example, in the gut, resident Treg cells help to prevent an uncontrolled response against the microbiome and food antigens, express the transcription factor FOXP3, sense vitamins and short‐chain fatty acids (SCFAs) and synthesise the anti‐inflammatory cytokine IL‐10 blocking the differentiation of T cells into pro‐inflammatory phenotypes, such as Th1 and Th17.^17^

Interestingly, the importance of the metabolic phenotype in the progression of autoimmune disorders has been recently shown, where reversing the CD4+ T‐cell abnormal phenotype contributes alleviating the severity of Hashimoto's thyroiditis (HT). Deactivation of the mTOR/HIF‐1α/glycolysis pathway in thyroiditis patients decreases the ratio of Th1 and Th17 phenotypes in favour of the Treg phenotype.^18^ Also, lower levels of the histone deacetylase (HDAC) Sirtuin SIRT1 are associated with altered T‐cell differentiation and elevated production of anti‐thyroid antibodies.^18^

Altogether, targeting glycolysis and/or ROS production and boosting the population of Treg cells may be a potential therapeutic strategy to inhibit autoreactive T‐cell activation, increasing self‐tolerance, and thus, prevent the development of autoimmune disorders.^19^

## 3 B‐CELL METABOLISM IN HEALTH AND DISEASE

In addition to the increasing evidence that T‐cell metabolic switches are involved in the development of immune disorders, the importance of B‐cell metabolism in (auto)immune disorders is emerging.^20^ The main connections between signalling and metabolic pathways of B cells in health and disease are indicated in Table 1. B cells require mechanisms to allow fast switching between high and low levels of metabolic activities depending on the stage of their life cycle. For example, proliferating precursors of B lymphocytes (pre‐B cells) and GC B cells during clonal expansion require high energy levels and availability of building blocks to carry out their function and development.^21^ Therefore, they rely on high levels of glycolytic flux. Conversely, quiescent naïve and long‐term memory B cells rely on fatty acid oxidation and OXPHOS, as they require lower metabolic demands. These cells have access to fewer survival and growth signals, relying on autophagy to meet the metabolic demands.^22^ Also, B cells exhibit a close correlation between different cellular and metabolic phenotypes. For example, hypoxia plays an essential role in coordinating the adaptation and function of immune cells. During their development, B cells are exposed to different oxygen levels that contribute to their activation and differentiation; HIF‐1 activation affects B‐cell metabolism, proliferation, apoptosis, antibody class switching and affinity maturation, and regulates their migration to and egression from lymphoid tissues.^23^, ^24^

**FIGURE 3:**
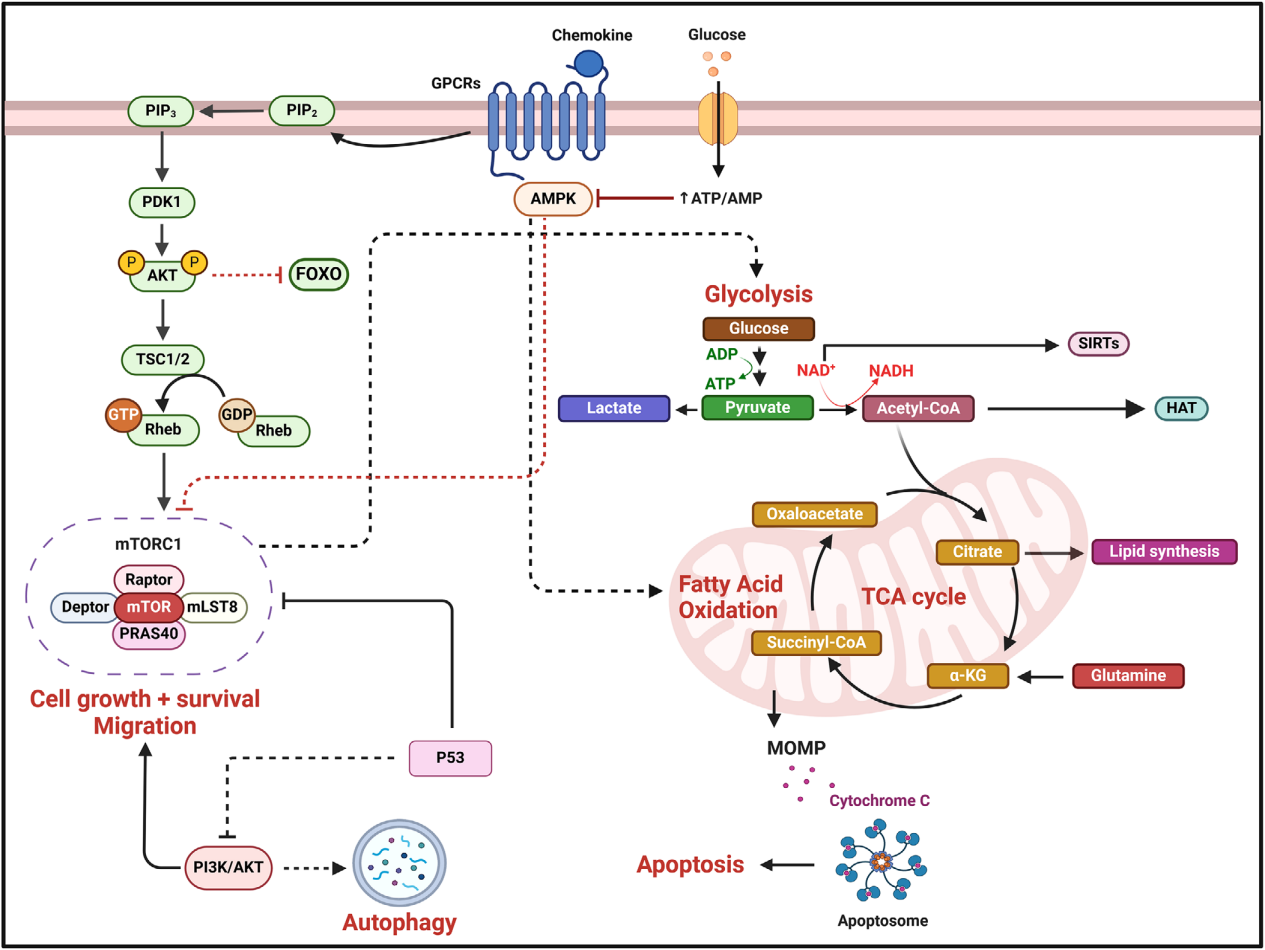
Metabolic and signalling processes underlying the activation of B (and T) cells. Proper stimulation of B‐ (and T‐) cell receptors and the metabolic state of the cell support immune cells through the various proliferation stages. Depending on the signals received, either metabolites or other molecular messengers, a cell triggers a series of signalling reactions affecting cell fate. For instance, glucose uptake results in a series of pathways, such as glycolysis and TCA cycle, being activated. Specifically, it inhibits the AMP‐activated protein kinase (AMPK) pathway, while the stimulation of chemokine receptors (as well as cytokine receptors, not shown) triggers the PI3K pathway, activating mTOR, which favours the flux through glycolysis. In addition, glucose uptake promotes controlled levels of autophagy to facilitate the recycling of molecules. Depending on the glycolytic flux, the excess of carbon source can be channelled from the TCA cycle into lipid synthesis via citrate production. Altogether, these metabolic processes are linked to activation of B (and T) cells, as they promote cell growth, survival and migration. However, in the absence of enough glucose (or other energy sources), the AMPK pathway stimulates fatty acid oxidation, additionally inhibiting the mTOR pathway and, thus, the glycolytic flux. Acetyl‐coenzyme A (Acetyl‐CoA) from lipid catabolism, as well as amino acids such as glutamine from protein degradation, can be fed into the TCA cycle to maintain the oxidative phosphorylation. This setting is often observed in quiescent cells, such as naïve T and B cell, or Treg cells, and in general for cells with low metabolic demands. If the energy demand increases without proper stimulation of pathways around mTOR, which allows for glucose uptake and fast energy production through glycolysis, autophagy will occur to compensate for the lack of nutrients; eventually, p53 will promote arrest of cell growth and survival signals. The stress on the mitochondria will then cause the permeabilisation of their membrane that can ultimately lead to cell death through apoptosis. Created with and adapted from BioRender.com.

In addition to the cell phenotype as well as growth and environmental factors, the signalling context in the form of cytokines and chemokines also affects the metabolic state of B cells. For example, IL‐4 increases glucose uptake in a PI3K‐independent manner, thus directing B cells to trigger the metabolic switch required for proliferation and survival. Without proper cytokine stimulation, B cells cannot control the oxidative stress derived from an increased metabolic demand, ultimately triggering the mitochondrial (intrinsic) apoptosis pathway. In this context, the activation of the (AMP‐activated protein kinase (AMPK) pathway is required for both the metabolic switch upon B‐cell receptor (BCR) stimulation and the activation of autophagy to meet metabolic demands.^22^ A summary of the main metabolic and signalling pathways involved in B‐ (and T‐) cell modulation is shown in Figure 3.

Similar to T helper (Th) cells, B‐cell phenotypes exist that have regulatory functions. For example, regulatory B cells (Bregs)—consisting of the 0.5% of human B cells from the periphery that expands after activation—have a negative immune regulatory role and lack a clearly defined phenotype, though they can induce the Treg phenotype in T cells. These cells appear to have anti‐inflammatory activity, helping to clear the consequences of an infection, and seem to be associated with metabolic reprogramming. Bregs produce the anti‐inflammatory cytokine IL‐10 through the phosphatidylinositol 3‐kinase /protein kinase B/glycogen synthase kinase‐3 (PI3Kδ/Akt/GSK3) cascade, which is under the control of cholesterol metabolism,^25^ as well as suppress the differentiation of inflammatory cell phenotypes, such as Th1, Th17 and natural killer T (NKT) cells.^26^

In the disease context, the effectiveness of treatment of the highly aggressive tumour diffuse large B‐cell lymphoma (DLBCL) with histone deacetylase inhibitors (HDACis) correlates negatively with high OXPHOS levels of B cells. Though the molecular mechanisms have not been elucidated yet, it has been recently shown that DNA phosphorylation and hyperacetylation promoted by HDACi trigger the activation of DNA repair pathways, which cause oxidative stress. Thus, cells carrying higher levels of OXPHOS can cope better with the stress promoted by HDACi drugs.^27^ The metabolism of B cells also impacts the development of different autoimmune disorders. For example, treatment of MS with Cladribine causes T‐ and B‐cell cytotoxicity and selectively depletes long‐term B‐cell populations. This drug affects the enzyme deoxycytidine kinase (DCK) involved in nucleotide metabolism, thus preferentially targeting B cells that undergo rapid proliferation, such as GC B cells, but not peripheral blood cells.^28^ Also, B‐cell populations are relevant to bone remodelling metabolism in RA, with B cells contributing to the inflammation of synovial tissues, through the production of the pro‐inflammatory cytokine tumour necrosis factor‐α (TNF‐α), via dopamine receptor 2 (DR2) signalling. DR2 expression correlates with TNF‐α production and can predict the response towards RA treatment.^29^

Another example of the relation between oxidative stress and B cells in disorders is the production of type I IFN in SLE that leads to mitochondrial dysfunction and ROS production affecting the metabolic environment. Deficient mitochondrial function results in the release of cell death‐inducing factors from the mitochondria into the cell, ultimately being capable of triggering cell death pathways, such as the apoptosis cascade. ROS abundance, in turn, can affect macromolecules, in particular proteins and lipids of the cell membrane. Therefore, damaged mitochondria should be cleared from cells. If the clearance process is disrupted, the mitochondrial DNA (mtDNA) in the cytoplasm can activate nuclear sensing pathways, including cyclic guanosine monophosphate‐adenosine monophosphate (GMP–AMP) synthase (cGAS) and Stimulator of Interferon Genes (STING) pathways (cGAS‐STING), promoting type I IFN synthesis.^30^ Altogether, this oxidative and inflammatory process enhances B‐cell differentiation into plasmablasts, with an undesired production of auto‐antibodies characteristic of SLE.^30^ Thus, strategies to promote mitochondrial functionality and reduce oxidative stress may contribute to immune tolerance and, therefore, may contribute to the effectiveness of treatments for autoimmune disorders such as SLE.

Memory B cells also rely on metabolic signalling to perform their functions and potentially become long‐lived cells.^31^ For example, the transition from an effector to a memory B‐cell phenotype requires high levels of adenosine triphosphate (ATP) and nicotinamide adenine nucleotide (NADH), achieved through an increase in glucose uptake and glycolytic flux. In this regard, overexpression of the B‐cell activating factor (BAFF) of the TNF family, which activates mTORC1 signalling, has been reported in several autoimmune disorders, such as RA and SLE.^32^, ^33^ B‐cell overexposure to BAFF, along with Myc expression, results in an upregulation of enzymes involved in glucose metabolism, i.e. glycolysis and OXPHOS, promoting cell survival and proliferation of potentially self‐reactive cells (Figure 2B). In addition, the survival of long‐lived cells is dependent on the tight regulation of autophagy.^22^ Unlike B cells in the GC, those in the peripheral tissues have limited access to nutrient and growth factors; signalling through the AMPK pathway is essential to balance the anabolic and catabolic processes, and in case of need, autophagy is activated to maintain the cellular energy levels.^22^, ^31^

## 4 T–B AND B–T CELL METABOLIC INTERACTIONS FOR CELL HOMEOSTASIS

Both T and B cells tightly regulate cell proliferation through the cell cycle; depending on the stage of the immune development or the immune response, the cell needs to rapidly switch between resting (metabolically inactive) and proliferative (metabolically active) states. The main connections between signalling and metabolic pathways of B–T/T–B cell interactions in health and disease are indicated in Table 1. A key regulator of this switch is the transcription factor Myc, which can modulate the proliferation rate: when Myc overcomes a critical threshold, T and B cells activate their metabolism by switching to glycolysis and then divide. Furthermore, the anti‐apoptotic protein Bcl‐2 senses the metabolic state of the cell: a decrease in its expression, when Myc production decreases, results in the release of pro‐apoptotic proteins that trigger the intrinsic or mitochondrial apoptosis pathway.^34^ In addition, Myc induction of amino acid transport is important for the biosynthetic programs that shape T‐cell phenotypes.^35^ Altogether, a tight regulation of the immune response integrates signals from the immune and metabolic pathways that control cell fate at both the transcription and metabolic levels.

The crosstalk among immune signalling, metabolism and the cell cycle calls for cellular molecules that may integrate these signals to guarantee cellular homeostasis, through sensing the metabolic state of the cell as well as the integrity of cellular structures. An example of such molecules are the Sirtuins, which are NAD+‐dependent proteins that connect metabolism and cell cycle; they are involved in the post‐translational modification of key metabolic signalling proteins, such as the kinases AMPK, PI3K, mTOR, cyclin‐dependent kinases (CDKs) and the transcription factors forkhead box (FOX). Therefore, it may be speculated that Sirtuins coordinate the metabolic changes required for the proliferation and differentiation of T and B cells with the changes required to allow for cell cycle progression, thereby cell proliferation.^36^

In the context of the prompt response of an organism to an insult, activation and proliferation of both T and B cells precedes the occurrence of T–B and B–T cell interactions, which are then essential for an appropriate immune response and immune cell homeostasis. Different T‐cell phenotypes can contribute to B‐cell activation, immunoglobulin class switching and posterior differentiation to either short‐lived or long‐lived memory cells or plasma cells. B‐cell maturation at the GC is indeed dependent on interactions with T cells, which allow their proliferation, somatic hypermutation, and the ultimate selection of high‐affinity B cells that will then produce high‐affinity antibodies. This activatory interaction, accompanied by the corresponding T‐cell‐dependent cytokine environment at the system level, stimulates B cells to differentiate into memory cells and, eventually, to plasma cells.^37^ In turn, B cells can act as APCs and drive CD4+ Th cell differentiation into different phenotypes, as well as reinforce the stimulation of CD8+ T cells.^38^ These T–B and B–T cell interactions have an impact on the metabolism, with relevance in a disease context.

Interactions between T and B cells are key for appropriate antibody production; therefore, understanding the events leading to and triggered T–B cell signalling is important when addressing autoimmune disorders. For example, the co‐stimulatory molecules CD40L and inducible costimulator (ICOS) on CD4+ T cells play a critical role in T–B interaction for B‐cell activation and differentiation. The blockade of ICOS dramatically reduces the adhesion between T and B cells, while blocking CD40L does not affect the interaction but leads to a dramatic decrease in IgM/IgG production, typical of SLE.^39^ Although the role of these co‐stimulatory molecules differs, they can both contribute to disease pathogenesis: while upregulation of ICOS causes prolonged delivery of help from CD4+ T cells to B cells, CD40L appears to be essential for correct B‐cell differentiation and antibody production.

Another example of T–B interactions is given in the context of inflammation and metabolic disorders, with a crosstalk between the central axis of the PI3K/Akt/mTOR pathways and the redox homeostasis integrated in the mitochondria. This is clearly observed in SLE, where mitochondrial dysfunction in T cells causes the release of pro‐inflammatory lipid hydroperoxides, depleting glutathione (GSH) from the cell and triggering activation of the PI3K/Akt/mTOR axis. The release of pro‐inflammatory cytokines contributes to B‐cell activation, in addition to the activated T‐cell‐mediated induction of B‐cell maturation, class switching and production of IgG antibodies, which are heavily involved in SLE pathogenesis. Given the involvement of oxidative stress in the pathogenesis of autoimmune disorders, in particular SLE, attempts have been made using antioxidant treatments to supplement SLE therapies. Although there is currently no evidence that supplementation with antioxidants ameliorates SLE symptoms,^40^ other drugs targeting the metabolism have been tested. Such an example is the bis‐2‐(5‐phenylacetamido‐1,3,4‐thiadiazol‐2‐yl) ethyl sulphide (BPTES). It is a selective inhibitor of the enzyme glutaminase 1 (Gls1), which converts glutamine to glutamate, that was administered to treat SLE‐like disease in mice. This treatment reduces HIF‐1α production, downregulating the glycolytic flux, thus ameliorating disease severity.^41^ A similar effect is shown upon simultaneous administration of metformin and the glucose metabolism inhibitor 2‐deoxy‐d‐glucose.^41^

An example of B–T interactions in the disease context is given by B cells IgG production and Th phenotype modulation that impact on glucose metabolism. Specifically, B‐cell‐mediated differentiation of the Th1 phenotype and the activation of effector CD8+ T cells, in addition to an enhanced IgG production, target islet cells causing insulin resistance and can lead to chronic inflammation.^6^ Similarly, B‐cell leukaemia affects T cells and metabolism. Specifically, B cells hyperactivate T cells with an increased expression of the programmed cell death protein 1 (PD‐1), reducing the inflammatory response, and leading to T‐cell exhaustion. Although the exact mechanism is currently not clear, T cells in (acute or chronic) B‐cell leukaemia exhibit a decreased mTOR/Akt signalling, which—consistently to what we discussed earlier—reduces glucose uptake via suppression of the glucose transporter Glut1 and increases the frequency of Treg cells.^42^ Similarly, lower levels of Glut1, deteriorated OXPHOS and overall reduced mitochondrial fitness are also observed in chronic lymphocytic leukaemia (CLL).^43^

Of note, it has been recently shown that the use of Burton's tyrosine kinase inhibitors (BTKi) serves as a potential treatment against MS. Specifically, inhibition of BTK limits B–T cell interactions through inhibition of B‐cell mitochondrial respiration.^44^ The reduction of mitochondrial respiration correlates with the downregulation of B‐cell pro‐inflammatory response, thus decreasing the ability of B cells to present antigens to T cells. Although BTKi limits mitochondrial respiration, thereby energy production, it does not impact on T‐cell/B‐cell survival, thus reducing undesired side effects.

Altogether, we highlight that targeting T‐ and B‐cell interactions at the metabolic level may be promising strategies to target pro‐inflammatory B cells for the treatment of autoimmune disorders. We therefore propose that the combination of treatments targeting immune cells that cause (T/B) cell phenotype imbalances along with metabolic switches involved, may increase the effectiveness of treatment of autoimmune disorders.

## 5 METABOLIC–EPIGENETIC REGULATION IN LYMPHOCYTE PHENOTYPE SWITCHING

Changes at the signalling and metabolic levels regulate gene expression, allowing for complete phenotype switches of lymphocytes. This regulation is mediated by epigenetic mechanisms that mark the DNA in response to environmental cues. Metabolic changes can affect the modification patterns of the chromatin, such as DNA methylation and histone acetylation, therefore contributing to the switch among immune cell phenotypes. In turn, epigenetic modifications may affect the expression of metabolism‐related genes, generating a feedback loop between metabolic and epigenetic changes.

**FIGURE 4:**
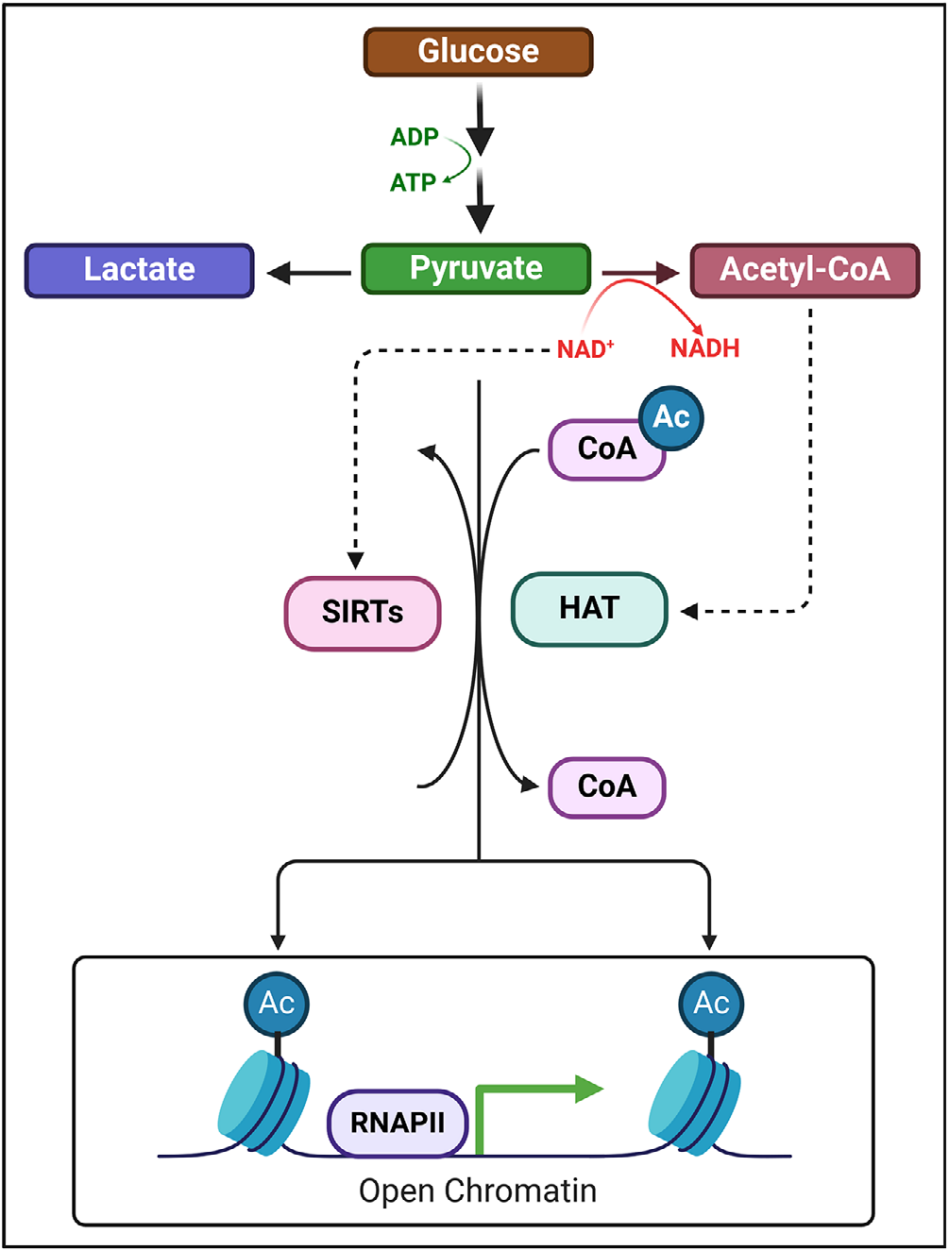
Connection between metabolism and epigenetic regulation. Metabolites function as signals between metabolism (in the cytoplasm) and epigenetic regulation (in the nucleus). For example, acetyl‐coenzyme A (acetyl‐CoA) measures the metabolic state of a cell, and it is involved in the acetylation of histones. Histone acetylases (HATs) use acetyl‐CoA as the acetyl group donor to modify histones, favouring the opening of chromatin, thus directly promoting gene expression. In turn, the expression of genes related to metabolic enzymes affects the flux through metabolic reactions. Conversely, Sirtuins (SIRTs), histone deacetylases which use NAD+ as a cofactor, promote the compaction of chromatin depending on the cellular NAD+/NADH ratio: during low metabolic states (more NAD+ than NADH), SIRTs will reduce transcription. A similar mechanism occurs for other metabolites, for example, S‐adenosyl‐methionine (SAM) in DNA methylation (not shown). Created with and adapted from BioRender.com.

Availability of metabolic cofactors modulates the crosstalk between metabolism and epigenetics in in lymphocytes. Metabolites such as S‐adenosyl methionine (SAM), acetyl‐coenzyme A (acetyl‐CoA), NAD, α‐ketoglutarate (αKG) and ATP serve as indicators of the metabolic state of the cell and are also cofactors for chromatin‐modifying enzymes. The feedback between expression of metabolic enzymes and epigenetic modifications allows for the rewiring of this ‘metabolic–epigenetic axis’. HDACs or Sirtuins such as Sirt1 regulate chromatin modifications in response to metabolic changes and signalling pathways (Figure 4). For example, Sirt1‐mediated deacetylation of STAT3, downstream mTOR activation, has an important role in Th17 differentiation; Sirt1 activators hinder the Th17 inflammatory phenotype, decreasing the expression of IL‐17A and the retinoic acid receptor‐related orphan nuclear receptor gamma (RORγt).^45^ Sirt1 also inhibits the c‐Jun transcription factor that induces IL‐2 production, decreasing Th1 activation, whereas Sirt4 inhibits AMPK signalling, suppressing Treg activity.^45^ Sirtuins’ activity can have pro‐ and anti‐inflammatory effects depending on the surrounding signalling context, highlighting the importance of the metabolic–epigenetic axis in the immune response.

The metabolic–epigenetic axis is critical also in B‐cell activation. On the one hand, BAFF is expressed in activated B cells, promoting the production of the anti‐inflammatory cytokines IL‐10 and IL‐35, and increasing B‐cell metabolic capacity by promoting glycolysis and mitochondrial OXPHOS through the PI3K/Akt pathway.^46^ On the other hand, B‐cell subsets expansion is regulated by histone modifications, DNA methylation patterns and the expression of non‐coding RNAs. DNA methyl transferases use SAM as a cofactor, further linking metabolism and epigenetic regulators. Furthermore, B cells rely on epigenetic mechanisms to increase their diversity; the action of the activation‐induced cytidine deaminase induces DNA breaks that promote B‐cell epigenetic diversity.^46^

Dysregulation of the metabolic–epigenetic axis can contribute to the pathogenesis of autoimmune disorders. HDAC dysregulation, together with hypermethylation of certain genes, is observed in Grave's disease (GD)—an autoimmune thyroid disease (AITD), in RA and osteoarthritis (OA). The modulation of HDAC such as Sirt1 reduces the expression of the transcription factor FOXP3 in T cells, reducing the prevalence of the Treg phenotype.^47^ Therefore, drugs such as HDACi and methotrexate (MTX) (promotes DNA demethylation) exhibit an anti‐inflammatory activity. Abnormal methylation patterns have been observed in different autoimmune disorders, such as MS, RA and SLE. In the latter, global hyper‐ or hypo‐methylation of genes related to arachidonic acid and homocysteine metabolic pathways are correlated with overactive B‐ and T‐cell responses.^48^ Arachidonic acid is a fatty acid and the precursor of numerous signalling lipids, highlighting the importance of lipid metabolism in the homeostasis of histone modification; most of the acetyl‐CoA used for histone acetylation derives from fatty acid oxidation, thus allowing a tight intercommunication between epigenetic modifications and lipids metabolism.^48^

An association between aberrant epigenetic marks and autoimmune disorders has been shown for both type 1 and type 2 diabetes (T1D and T2D). In T1D, hypermethylation of FOXP3 promoter in CD4+ T cells is associated with the destruction of pancreatic β‐cells.^49^ In T2D, patients with an increased rate of DNA methylation in genes deregulating β‐cell functions, such as PDX1, PPARGC1A and GLP1R, may exhibit affected pancreatic cell development, energy metabolism and insulin secretion, respectively, increasing the risk of developing the disease.^49^

Finally, it has been recently observed in a mouse model that itaconate, metabolite derived from the TCA cycle and correlated with the inhibition of synthetic enzymes and the reduction of the SAM/S‐adenosylhomocysteine (SAH) ratio, inhibits Th17 differentiation while promoting the Treg phenotype, inhibiting glycolysis and OXPHOS.^50^ This inhibition does not occur through blocking the HIF‐1α/mTOR pathway, but through affecting the chromatin accessibility in specific genes; specifically, itaconate inhibits the production of IL‐17 and granulocyte macrophage colony‐stimulating factor (GM‐CSF) in pathogenic Th17 cells, while promoting FOXP3 expression in Treg cells.^50^

Altogether, evidence is progressively accumulating about the relevance of the metabolic–epigenetic axis in the development of autoimmune disorders. The mutual interplay between these regulatory layers contributes to the phenotype switching of T and B cells, together with specific inflammatory and metabolic signalling, thus highlighting the cellular inter‐communication underlying systemic diseases.

## 6 AGEING CROSSING AUTOIMMUNE AND METABOLIC DISORDERS

Altogether, the information presented so far suggests that, when targeting an immune disorder, the effect it may have on the metabolism shall be considered. An intricate relationship between the immune response and metabolic changes exists, with metabolic resources required to support the immune system‐mediated organismal response to an insult, and with immune imbalance affecting the organism's metabolic state. Indeed, chronic inflammation and metabolic syndrome are closely related.^3^ Recent evidence shows an association of aged T and B cells to dysfunctions of mitochondrial metabolism and energy production,^51^ with defective mTOR pathway in T cells and defective Sirtuins–Forkhead transcription factor axis in B cells that also regulates cell cycle dynamics.^52^ The metabolic switch from a high glycolytic metabolism to defects in the mitochondrial metabolism, through an increase of the mTOR pathway in aged T cells and a decrease in the expression of SIRT1 and FOXO1 genes in aged B cells, may contribute to ageing of the adipose tissue and result in the inflammation characteristic of obesity and metabolic disorders.^2^, ^3^

Considering age along with immune and metabolic disorders provides a ‘bigger picture’ where T–B cell interactions are central in tackling systemic diseases of the immune system. The risk of metabolic disease increases with age, as macrophages release pro‐inflammatory signals and activate the NLRP3 inflammasome pathway that drives metabolic dysfunction.^3^ This leads to an elevated ROS production, a decrease in the NAD+/NADH ratio, and a suppression of SIRT2 in senescent T cells. These cells are unable to proliferate, activate either p53/p21 or pRb/p16 cell cycle pathways, and produce high levels of senescence‐associated secretory products (SASPs) such as IL‐1β and IL‐6. Subsequently, IL‐1β inhibits B‐cell lymphopoiesis—the process of generation of B lymphocytes—while promoting the differentiation of B cells to plasma cells.^2^ Altogether, differentiated phenotypes of T and B cells predominate, with naïve populations being reduced, and T–B cell interactions are dysregulated.^53^ Memory T and B cells tend to accumulate as tissue resident in peripheric organs, potentially recognising an infection in non‐lymphoid organs and activating a stronger and faster defensive response compared to that of their circulating counterparts, thus leading to a pro‐inflammatory state.^53^, ^54^

Ageing is associated to an increased inflammation, with most autoimmune disorders developing or worsening with age. Indeed, T‐cell ageing‐associated phenotypes (TASPs) are observed in T cells of RA patients. These cells are characterised by shorter telomeric DNA and instability of mtDNA, thus being more prone to release pro‐inflammatory effector cytokines. TASPs are more invasive and activate the inflammasome, leading to T‐cell death via pyroptosis, a death form that is strongly inflammatory.^55^ Furthermore, a decrease of new T cells due to the bone marrow decline and thyme involution is observed, with damaged molecules accumulating, and cells entering the senescence program. TASPs are characterised by gain and loss of function features, resulting in a hybrid phenotype, and aged T cells transition from stable highly specific phenotypes to more ‘innate‐like’, short‐lived inflammatory phenotypes.^55^ Paradoxically, these cells have a reduced capacity to respond to infections and immunisations but an increased probability to develop autoinflammatory and autoimmune disorders.

Because metabolic imbalances can cause or potentiate autoimmune disorders, and these are more likely to be developed with age, it is critical to investigate the relation between cellular senescence and metabolic changes. Recent evidence shows that obesity, a metabolic disorder, appears to accelerate age‐associated defects observed in human B cells on top of promoting chronic inflammation.^56^ Specifically, the fatty acid palmitate can induce a metabolic reprogramming of the glycolytic metabolism necessary for B‐cell expansion.^56^ A chronic increase in circulating levels of palmitate is observed, due to an increased lipolysis occurring during ageing and obesity, that leads to the activation of autoreactive B cells and induces immune senescence. Also, oxidative stress is increased in obesity, causing increased lipid peroxidation, and is an early instigator of metabolic syndrome.^57^

Thus, age reduces adaptative immunity and increases the risk of developing metabolic and autoimmune disorders, resulting in a less efficient response against new pathogens.

## 7 TARGETING METABOLISM IN AUTOIMMUNE DISORDERS: A PERSPECTIVE

In recent years, the concept of targeting metabolism to treat autoimmune disorders has become increasingly attractive. Generally, metabolic inhibitors target preferentially autoreactive cells, as they will affect proportionally more cells with aberrant metabolic states. Glucose transporter inhibitors block glycolysis in CD4+ T cells, decreasing their activation, GC response, and autoantibody production in SLE patients and in mice models of the disease.^58^ Also, sirolimus (mTOR inhibitor) serves as a treatment for lupus nephritis when the standard therapy is not tolerated.^58^ Moreover, targeting lipid metabolism ameliorates the disease. On the one hand, targeting cholesterol efflux with liver X receptor (LXR) agonists may help to balance lipid biosynthesis in dyslipidaemia that is highly prevalent in SLE patients.^58^ Cholesterol, together with phospholipids and glycosphingolipids contained in lipid rafts in cellular membranes coordinate cell signalling through different cellular routes such as the PI3K pathway. On the other hand, dietary SCFAs can attenuate SLE by regulating B‐cell differentiation.^59^ Also, because fatty acid synthase (FASN) downstream acetyl‐CoA carboxylase promotes inflammatory Th17 cells, inhibition of FASN reduces IFN‐γ production by Th1 and Th1‐like Th17 cells, leading to disease reduction.^60^

Despite different metabolic and signalling pathways have been mentioned so far, the best‐characterised metabolic pathway is the glycolysis. The regulation through the Akt/PI3K/mTOR signalling cascade is widely studied, with definite targets at both genetic and metabolic levels that make them attractive for therapies. Indeed, targeting glycolysis via mTOR ameliorates symptoms of autoimmune disorders, for example, improving disease markers such as the SLE activity index in both humans and mice models. Also, glucose metabolism has an essential role in T‐ and B‐cell development and activation. For example, deficiency of the glycolytic enzyme 6‐phosphofructo‐2‐kinase/fructose‐2,6‐bisphosphatase isoenzyme 3 (PFKFB3) in RA impairs redox balance, autophagy and decreases glycolysis, thus decreases ATP generation in naïve T cells, which instead exhibit increased activity of the pentose phosphate pathway (PPP) and NADPH production.^61^ Examples of other metabolites that may be relevant for targeting purposes being regulators of immune response and chronic inflammation are lactate (i.e., the rate of lactate production), and the redox regulation of GSH via transaldolase in the PPP. Overall, an increased oxidative stress is observed in T cells from lupus mice models.

Besides glucose/carbohydrate‐related cascades, metabolic changes in the disease context have been observed in other pathways, such as a higher catabolism rate of lipids in the mitochondria.^62^ Lipids are ideal therapeutic targets because they act as an energy source, constituting the main building block of cellular membranes, and their synthesis is increased during immune cell's activation. For example, the regulation of mevalonate pathways and fatty acid binding proteins (FABPs) are needed for the proliferation of Treg cells. Inhibition of FABP5 triggers activation of the cGAS‐STING pathway, inducing IL‐10 production and promoting the inhibitory activity of Treg, thus attenuating autoimmune disorders.^62^ In RA, an enhanced phospholipase A2 activity, which promotes changes in the structure of membrane lipid rafts, is observed, whereas n‐3 polyunsaturated fatty acids (PUFAs) seem to relieve disease symptoms, suggesting that diet supplementation can improve the efficacy of treatments, as well as contribute to ameliorating disease symptoms.^62^ Furthermore, autoimmune disorders can be suppressed by anti‐inflammatory bioactive lipids such as lipoxin A4 (LXA4), through inhibition of the cGAS‐STING pathway.^63^

Amino acid metabolism provides factors involved in cell metabolism, protein translation and proliferation. Glutamine maintains T‐cell function through TCA metabolism, where αKG affects the Th1/Treg ratio during T‐cell differentiation.^62^ Also, leucine regulates mTOR activity, promoting differentiation of the effector phenotypes Th1, Th2 and Th17. Deficiency in leucine transporters such as SLC7A5, as well as a reduced uptake of leucine and other large neutral amino acids impairs T‐cell differentiation.^62^ Furthermore, T‐cell activation is regulated by tryptophan and arginine, whose inhibition can lead to impaired cell proliferation and increased apoptosis.^62^

Finally, cofactors and vitamins may be targeted to modulate the immune system, and thus, to treat autoimmune disorders. For example, vitamin D, which has a predominant role in calcium homeostasis, has immunomodulatory effects on T cells. Specifically, the vitamin D receptor (VDR)‐driven response occurs with the concomitant inhibition of inflammatory T cells, favouring tolerogenic phenotypes, whereas vitamin D deficiency may lead to dysregulation of T‐cell function and increase the risk of autoimmune disorders.^64^

Altogether, metabolic processes such as glycolysis, fatty acid synthesis, amino acid metabolism, mitochondrial OXPHOS and several others, regulate the immune cell fate. The identification of metabolic targets, among which those highlighted above, is crucial for development of new therapeutic treatments against inflammation in autoimmune diseases.

## 8 OUTLOOK

We have previously proposed that autoimmune disorders may develop as a consequence of a metabolic imbalance that modulates switching between T‐cell phenotypes.^1^ Here, in a systems immunology view, we move a step forward discussing available evidence to propose a new perspective of targeting T–B and B–T cell metabolic interactions for the treatment of autoimmune disorders. In this context, we also highlight the coordination among inflammation, immunometabolism, epigenetics and ageing as a novel route to tackle autoimmune and metabolic disorders that is in its infant stages of understanding.

**FIGURE 5:**
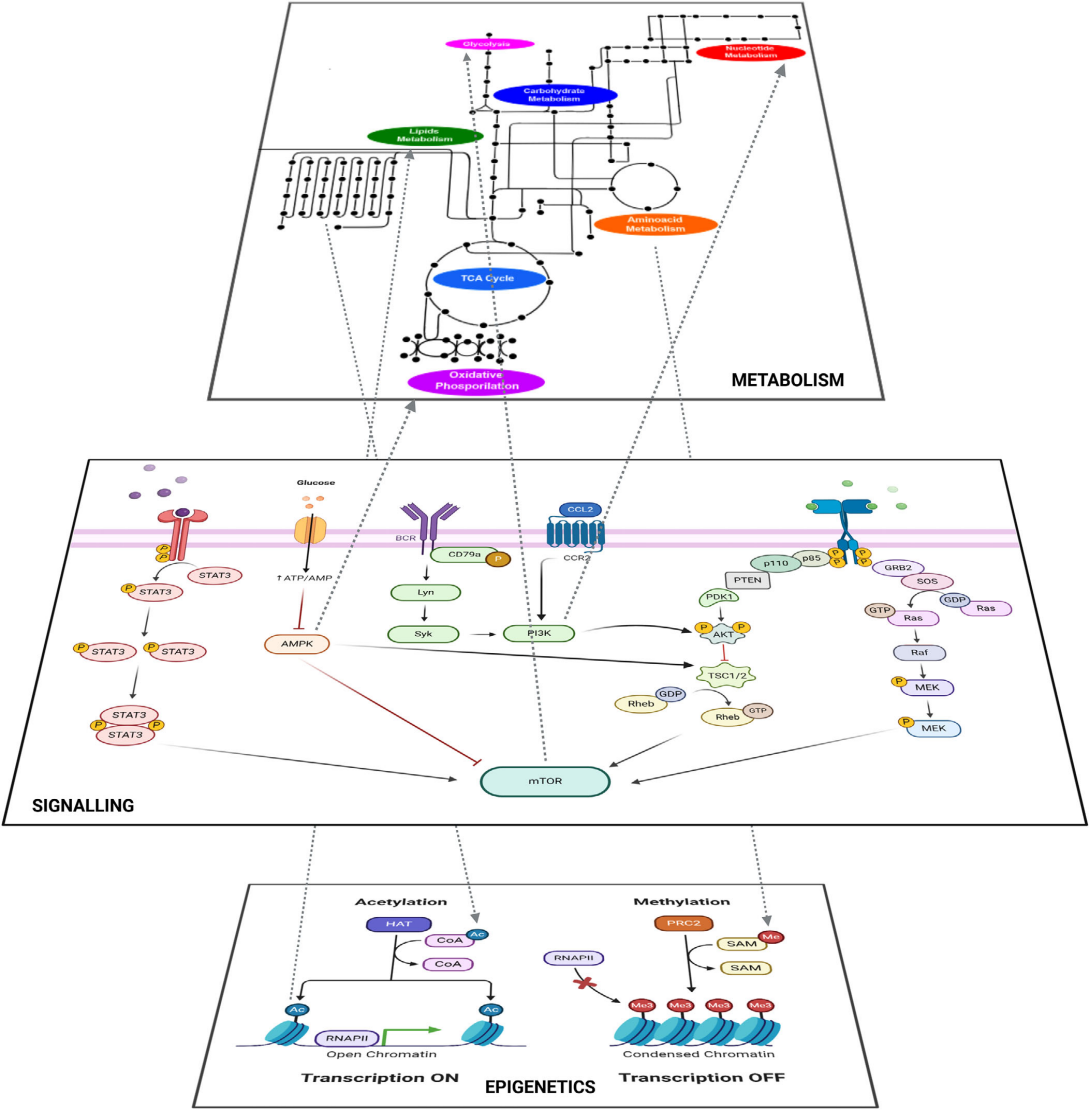
Interactions among three cellular layers involved in regulating B‐ and T‐cell phenotypes: metabolism, signalling and epigenetics. Activation of multiple signalling pathways such as mTOR or PI3K allows for the increase or decrease of the flux through multiple metabolic pathways, depending on the context. On the one hand, stimulation of AMPK promotes autophagy and oxidative phosphorylation (OXPHOS) in naïve or otherwise quiescent B or T cells. On the other hand, the activation of mTOR promotes the expression of the transcription factor hypoxia inducible factor 1α (HIF‐1α), therefore increasing the expression of the GLUT1 transporter and the rate of glycolysis on activated B and T cells. Also, PI3K activation can promote the activation of biosynthetic pathways required during cell proliferation, such as nucleotide synthesis. Furthermore, metabolism interacts with the two main epigenetic mechanisms: DNA methylation and histone acetylation, which require specific metabolic cofactors. Acetylation requires acetyl‐coenzyme A (acetyl‐CoA), which is mainly obtained from fatty acid oxidation, whereas methylation requires S‐adenosyl‐methionine (SAM), which is related to amino acid and cofactor metabolism. In addition, epigenetic modifications of the chromatin affect the expression of enzymes and signalling molecules involved in metabolic and signalling pathways. Created with and adapted from BioRender.com.

As the rationale of our reasoning, we have discussed the intricate relationship between the immune response and the underlying metabolic switches, with the immune cell‐mediated execution of the adaptive immune program requiring close cooperation of metabolic and signalling pathways. At the same time, we have provided hints that activation of autoreactive immune cells and aberrant inflammation may create an environment that favours the development of metabolic disorders. In this systems immunology context, continuous feedback exists between metabolic, signalling and epigenetic pathways (Figure 5). Thus, modulation of metabolism is not only desired but wanted, as different metabolic routes modulate T and B‐cell phenotypes that underlie pro‐inflammatory (disease) states occurring in autoimmune disorders. Therefore, treatment of an (auto)immune disorder may be not successful through the classical approach of employing drugs that target only an individual metabolic species at a time. Instead, although it may be challenging in many ways, we envision a combinatorial strategy that considers treatments targeting *at the same time* selected molecules in the immune pathways *and*—not *or*—in the metabolic pathways. This strategy can help to repurpose already existing drugs targeting the immune system and the metabolism for therapeutic treatment.

The role of metabolism in the progression of disease is not only confined to autoimmune disorders. Also, the inflammatory aspect of multiple diseases is critical regardless of whether its aetiology is immune. For example, in neurodegenerative diseases such as Alzheimer's disease (AD) where the inflammation is being triggered by oligomerisation of β‐amyloid (Aβ), the neurotoxicity is carried out by immune cells. Aβ accumulation misdirects the attack of immune cells towards neurons, causing necrotic cell death. In turn, the neural breakdown affects adjacent cells through the release of pro‐inflammatory molecules, resulting in the release of further Aβ, and generating a chronic self‐perpetuated autoimmune cycle.^65^ Analysis of clinical data has shown that decreased tryptophan levels correlate with poorer disease development and increased cognitive dysfunction, though the mechanism is yet to be elucidated.^65^ Thus, to target metabolism may be promising not only in immune disorders but in all diseases and conditions in which the ‘immune layer’ can be targeted.

## AUTHOR CONTRIBUTIONS

Matteo Barberis conceived and formulated the ideas and hypotheses, drawn the logic of the manuscript and figures and wrote the manuscript. Alejandra Rojas López contributed to the logic of the manuscript, helped with the initial drafting of figures and wrote the manuscript.

## CONFLICT OF INTEREST STATEMENT

The authors declare they have no conflicts of interest.

## ETHICS STATEMENT

No ethical approval was required.
